# Subpopulation of Macrophage-Like Plasmatocytes Attenuates Systemic Growth via JAK/STAT in the *Drosophila* Fat Body

**DOI:** 10.3389/fimmu.2020.00063

**Published:** 2020-01-31

**Authors:** Mingyu Shin, Nuri Cha, Ferdinand Koranteng, Bumsik Cho, Jiwon Shim

**Affiliations:** ^1^Department of Life Science, College of Natural Science, Hanyang University, Seoul, South Korea; ^2^Research Institute for Natural Science, College of Natural Science, Hanyang University, Seoul, South Korea; ^3^Research Institute for Convergence of Basic Sciences, College of Natural Science, Hanyang University, Seoul, South Korea

**Keywords:** plasmatocytes, *upd3*, *Drosophila melanogaster*, JAK/STAT, insulin signaling, *Hemolectin*, *Peroxidasin*

## Abstract

*Drosophila* hemocytes, like those of mammals, are given rise from two distinctive phases during both the embryonic and larval hematopoiesis. Embryonically derived hemocytes, mostly composed of macrophage-like plasmatocytes, are largely identified by genetic markers. However, the cellular diversity and distinct functions of possible subpopulations within plasmatocytes have not been explored in *Drosophila* larvae. Here, we show that larval plasmatocytes exhibit differential expressions of *Hemolectin (Hml)* and *Peroxidasin (Pxn)* during development. Moreover, removal of plasmatocytes by overexpressing pro-apoptotic genes, *hid* and *reaper* in *Hml*-positive plasmatocytes, feeding high sucrose diet, or wasp infestation results in increased circulating hemocytes that are *Hml*-negative. Interestingly these *Hml*-negative plasmatocytes retain *Pxn* expression, and animals expressing *Hml*-negative and *Pxn*-positive subtype largely attenuate growth and abrogate metabolism. Furthermore, elevated levels of a cytokine, *unpaired 3*, are detected when *Hml*-positive hemocytes are ablated, which in turn activates JAK/STAT activity in several tissues including the fat body. Finally, we observed that insulin signaling is inhibited in this background, which can be recovered by concurrent loss of *upd3*. Overall, this study highlights heterogeneity in *Drosophila* plasmatocytes and a functional plasticity of each subtype, which reaffirms extension of their role beyond immunity into metabolic regulation for cooperatively maintaining internal homeostatic balance.

## Introduction

The underlying mechanisms of the innate immune system of *Drosophila melanogaster* is paralleled in vertebrates (1). For example, *Drosophila* Toll receptor is functionally homologous to mammalian Toll-like receptors (TLRs) and their task to protect host from pathogens is conserved in vertebrates (1, 2). Also, *Drosophila* innate immune pathways which include the imd pathway, though having different NF-kB—*relish* for imd pathway and *dorsal* for Toll pathway—maintain comparable roles in host defense as in mammals (1, 3–5).

Comparable to vertebrates, hematopoiesis in *Drosophila* progresses in two waves: primitive and definitive hematopoiesis (6, 7). In the first wave or primitive hematopoiesis, hemocytes originate from the head mesoderm of embryo (8), and embryonically derived hemocytes comprise most circulating hemocytes during larval stages (9–11). However, not all hemocytes move freely within the hemolymph; a portion of embryonic hemocytes become localized at discrete regions within the larval cuticle called the hematopoietic pocket (12–14). Thus, the embryonic hemocytes become divided into two categories: circulating and sessile, depending on their mobility or locale within the hemocoel (12). At the hematopoietic pockets, resident hemocytes can be seen around oenocytes or neurons and their positioning is controlled by sensory neurons of the peripheral nervous system (14). Definitive hematopoiesis occurs during larval stages in the lymph gland, the hematopoietic organ of *Drosophila* larvae (7, 15). In the lymph gland, hemocytes are classified into four clusters: the posterior signaling center, the medullary zone, the intermediate zone and the cortical zone (16–18). Prohemocytes in the medullary zone progress through the intermediate zone and eventually differentiate into plasmatocytes, crystal cells or lamellocytes in the cortical zone (17, 18). During the pupal stage, hemocytes in the lymph gland dissociate and spread throughout the whole body, becoming the hemocytes of the adult fly (11, 19).

*Drosophila* hemocytes are largely recognized based on the expression of genetic markers throughout their development (20). Plasmatocytes comprise about 95% of the total hemocyte population and are functionally akin to mammalian macrophages (8, 21–23). They uptake pathogenic or cellular debris, and are marked by *Hemolectin* (*Hml*), *Peroxidasin* (*Pxn*), or *Nimrod C1* (*NimC1*) (20). Mature crystal cells are characterized by their internal crystalline structures and mediate melanization response to protect animals from injury or immune challenges. Crystal cells normally constitute about 5% of total hemocytes and are distinguished by the expression of *hindsight* (*hnt*), *lozenge* (*lz*), or *Prophenoloxidase* (*PPO*) 1 and 2 (9, 20, 23). While lamellocytes barely exist in healthy larva, they are differentiated from plasmatocytes in circulation or from the lymph gland in copious amounts upon immune challenges (12, 24–26). L1 (*atilla*), L2, L4, L6, or *misshapen* (*msn*) are used as markers for the lamellocytes (20, 27).

The JAK/STAT signaling cascade was first discovered in mammals where a variety of cytokines and growth factors transduce the signaling pathway related to immune responses (28). This pathway is highly conserved throughout evolution and is involved in critical biological processes of *Drosophila* including embryogenesis, immunity and stem cell maintenance (29). The JAK/STAT pathway in flies was originally highlighted in embryonic development where four main components are utilized: a ligand called *unpaired* (*upd*), a *domeless* (*dome*) receptor, the JAK—*Hoscotch* (*Hop*), and the *STAT* (30–32). In addition to the main players, negative regulators of the pathway have been also identified, including Socs36E, dPIAS, PTP61E or a BCL-6 homolog, Ken and Barbie (33). A role of JAK/STAT signaling in hemocyte development and immune responses was initially shown by a gain-of-function allele of *hop, hop*^*Tuml*^, which leads to hyperproliferation of hemocytes and formation of melanotic tumors (34, 35). Consistent with the hematopoietic phenotype observed in *hop*^*Tuml*^ mutants, active JAK/STAT signaling is required for differentiation of lamellocytes upon wasp infestation (36). Moreover, main players of the signaling such as *upd2* and *upd3* are upregulated in hemocytes upon immune challenges (37). During cellular immune responses, hemocytes induce *upd* ligands and secrete them to the hemocoel, where active propagation of JAK/STAT signaling in various tissues including the muscle, occurs. Amongst target tissues, the activation of JAK/STAT signaling in the muscle is linked to insulin signaling and carbohydrate metabolism, directly coupling immunity and metabolism (38). *Drosophila* fat body is the main source for antimicrobial peptides (AMPs), which facilitate the humoral immune response (37–39) as well as for the orchestration of metabolic events to maintain internal energy balance during feeding or non-feeding states (39, 40). Insulin production and secretion in the brain insulin producing cells (IPCs) is remotely controlled by the nutrient sensing from the fat body and vice versa, fat contents in the fat body is regulated by the insulin signaling (41, 42). Therefore, the mutual interactions between the insulin signaling and the fat body coordinate metabolism and growth of animals in response to availability of nutrition (41, 43, 44). Interestingly, recent studies have shown that active innate immunity attenuates growth and nutrient storage by blocking PI3K and AKT in the fat body, establishing an intricate balance between insulin signaling and innate immunity in the fat body (42, 45).

*Drosophila* hemocytes have been largely classified based on their morphology and expression of a few marker genes (20). Plasmatocytes constitute the largest population and show significant functional diversity; however, it is not clear whether the current classification sufficiently describes possible heterogeneity within plasmatocytes (1, 8, 12, 46). Moreover, the developmental fluctuations within plasmatocytes have not been examined at a cellular level. To investigate cellular discrepancies of circulating plasmatocytes in developing *Drosophila* larvae, we utilized two binary systems, Gal4-UAS and LexA-LexAop, to simultaneously visualize two representative markers, *Hml* and *Pxn* (47, 48).

In this study, we show that *Hml*-positive (*Hml*^+^) and *Pxn*-positive (*Pxn*^+^) plasmatocytes generally overlap in embryonically derived hemocytes. However, a subpopulation of plasmatocytes exhibit only Hml or Pxn expression distinctive from the double-positive (*Hml*^+^ *Pxn*^+^) plasmatocytes. Upon expression of pro-apoptotic genes, *hid* and *reaper* (*rpr*), in *Hml*^+^ hemocytes, *Pxn*-positive and *Hml*-negative (*Pxn*^+^*Hml*^−^) hemocytes increase accompanied by elevated levels of a cytokine, *unpaired 3* (*upd3*) in hemocytes. Interestingly, *upd3* from hemocytes activates the JAK/STAT signaling in various tissues including the fat body, which attenuates the insulin signaling pathway and leads to systemic metabolic dysfunction. Thus, identification of plasmatocyte subpopulations in this study enriches the concept of hemocyte heterogeneity and appends metabolism mediation to the role of plasmatocytes in immunity for the purpose of keeping internal homeostasis.

## Materials and Methods

### Fly Stocks and Genetics

Larvae and flies were generally reared in a *Drosophila* chamber which is maintained at 25°C and 70% humidity. To enhance *Gal4/UAS* and *LexA/LexAop* expression, larvae were shifted to 29°C after egg-laying. Also, they were cultured on normal food comprising of dextrose, cornmeal, dried yeast, and agar. Fly stocks used in this study include: *Hml*^Δ^*-Gal4, UAS-2XEGFP* (S. Sinenko), *Pxn-Gal4, UAS-GFP* (U. Banerjee), *UAS-hid, rpr* (Nambu J. R.), *UAS-upd3* (B. Lemaitre), *STAT92E::edGFP* (N. Perrimon), *Hml-Gal4* (U. Banerjee). *13XLexAop2-6XmCherry-HA* (BL52271), *LexAop-mRFP.nls* (BL29956) *UAS-Ras85D RNAi* (BL34619), *UAS-hid/Cyo* (BL65403), w^*^upd2^Δ^*upd3*^Δ^ (BL55729), *tGPH* (BL8164) were received from the Bloomington *Drosophila* Stock Center.

For high sugar diet, we substituted dextrose with sucrose (100 g/L; therefore, 300 mM). Synchronized first instar larvae were collected and transferred to high sucrose diet and kept at 25°C. We dissected or bled larvae at 72, 96, or 120 h AEL.

### Generation of *Pxn*-LexA and *upd3*-LexA Flies

Amplified *Pxn* enhancer from genomic DNA was cloned into TA-TOPO vector (Invitrogen, K252020) for gateway cloning (Primer-forward: CTCACCAACTGGATGTTGGTC/ Primer-Reverse: CCCAAACAAATATCTGTAGACTGACAG). Also, *upd3* enhancer was amplified for cloning into TA-TOPO vector (Primer-forward: TCGTACAATGGTTTAAAAATAGCTCGGCCAAAT/ Primer-Reverse: AGTGACCAGTTCCTGTTCAGGCGTCGTCGTCGAT). Cloned entry vector were ligated into destination pBPnlsLexA::p65Uw (Addgene, #26230) vector by using LR ligase II (Invitrogen, 11791-020). Recombinant constructs (at least 20 μg DNA) were injected into flies and generated by BestGene Inc.

### Immunohistochemistry

Larvae were dissected in late third instar stage in 1 × PBS, fixed in 3.7% formaldehyde (Sigma, F1635) in 1 × PBS for 30 min at room temperature and washed three times in 1× PBS containing 0.4% triton-X (1 × PBS-T) for 10 min each. For measurement of total hemocytes, larvae were vortexed for 2 min in 1 × PBS and hemocytes were allowed to ooze out for 30 min (except staining for L1 which was 1 h) on ice. Tissues were blocked in 10% normal goat serum (Vector Laboratories) in 1× PBS for 30 min. Primary antibody was incubated with tissue overnight at 4°C and then washed three times in 0.4% 1× PBS-T. Secondary antibody was incubated 3 h at room temperature. Samples were washed three times in 0.4% 1× PBS-T and mounted in Vectashield (Vector Laboratories, H-1200). Primary antibodies used in this study: rabbit anti-Pxn [1:1,000, (49)], mouse anti-P1 (1:100, Istvan Ando), mouse anti-L1 (1:100, Istvan Ando), mouse anti-Hnt (1:10, DSHB), mouse anti-nc82 (1:10, DSHB), rabbit anti-dFOXO (1:100, Yu. K), rabbit anti-DCP-1 (1:200, Cell signaling, 9578), Rho-phalloidin (1:100, Invitrogen) and BODIPY 493/503 (1:200, Molecular Probes, 3922). Cy3-, Alexa Fluor 647- and FITC-conjugated secondary antibody were obtained from Jackson ImmunoResearch Laboratories Inc., and each antibody was used at 1:250 dilution ratio.

### Imaging and Quantitation

All fluorescence was imaged by confocal microscopy (Nikon C2 si-plus). Two micrometers step Z stacks of larval brain, fat body, muscle, salivary gland, and intestine with identical laser power and scan setting were taken. Mean intensity of all images for each sample were calculated using Image J, Imaris (Bitplane), and Microsoft Excel software.

### Quantitative Real-Time PCR

For measurement of gene expression, we collected samples on ice: 70 blood volumes, 10 fat body, 25 brains, 10 muscles, 10 whole larvae. RNA was isolated from tissues by using Trizol (Invitrogen). cDNA was synthesized with RT kit (TOYOBO). RT-PCR was performed using SYBR Green master mix on a Step One-Plus Real-Time PCR thermal cycler (Applied Biosystems). Gene expression was normalized by *rp49*. Primers used for qPCR is tabled Supplementary Material 1.

### Measurement TAG in Fat Body

Collection of 10 fat body on ice with 0.05% 1 × PBS-T (Tween 20) and then homogenization of tissues with pestle (or rapidly kept in−80°C deep freezer until use). Homogenate tissues were heated on 70°C for 10 min to inactivate lipases and previous method was followed (50). For the measurement of TAG in samples, Serum triglyceride determination kit (Sigma, TR0100-1KT) and Glycerol standard solution (Sigma, G7793) were used. The samples were assayed using a plate reader to measure absorbance at 540 nm.

### Measurement of Pupa Volume

Animals were segregated into male and female groups per genotype at the larval stages. Upon pupariation, 20–50 pupae were arranged on a silicon pad and photographed using Nikon SMZ18 and ProgRes CapturePro v2.8.8 software. Length and diameter of pupa was obtained using ImageJ. Pupa volume was calculated as previously described (51) using Microsoft Excel. Prism8 was then used to determine *P*-values and generate final graphs.

### Measurement of Adult Weight

Adult fly mass was measured as previously described (52). Briefly, 1 day old adult flies were segregated by gender over CO_2_ anesthetic pads. Batches of 15–25 animals for each gender and genotype were collected onto a filter paper and placed on a sensitive balance (Ohaus Pioneer, PAG 214) to determine overall mass. The average mass of animals per gender and genotype was calculated with Microsoft Excel. Prism8 was then used to determine *P*-values and generate final graphs.

### Measurement of Pupation Time

Synchronized first instar larvae were collected in batches into a vial containing standard fly media and kept at 25°C. The number of larvae turning into pupa was counted against the transition time point. The number of larvae turned into pupa was then calculated as a percentage of the overall number of pupae in a vial for each time point using Microsoft Excel. Final graph was generated using Prism8.

### Wasp Infestation

Larvae were infested at 60 or 72 h AEL with *Leptopilina boulardi* for *Pxn*/*Hml* population count or *Ras85D* RNAi, respectively. Egg deposition was confirmed by direct observation of wasp eggs, after 8 h of co-culture. All infestation procedures were performed at 25°C.

## Results

### Distinctive Patterns of *Hml* and *Pxn* in the Larval Hemocytes

**Figure 1:**
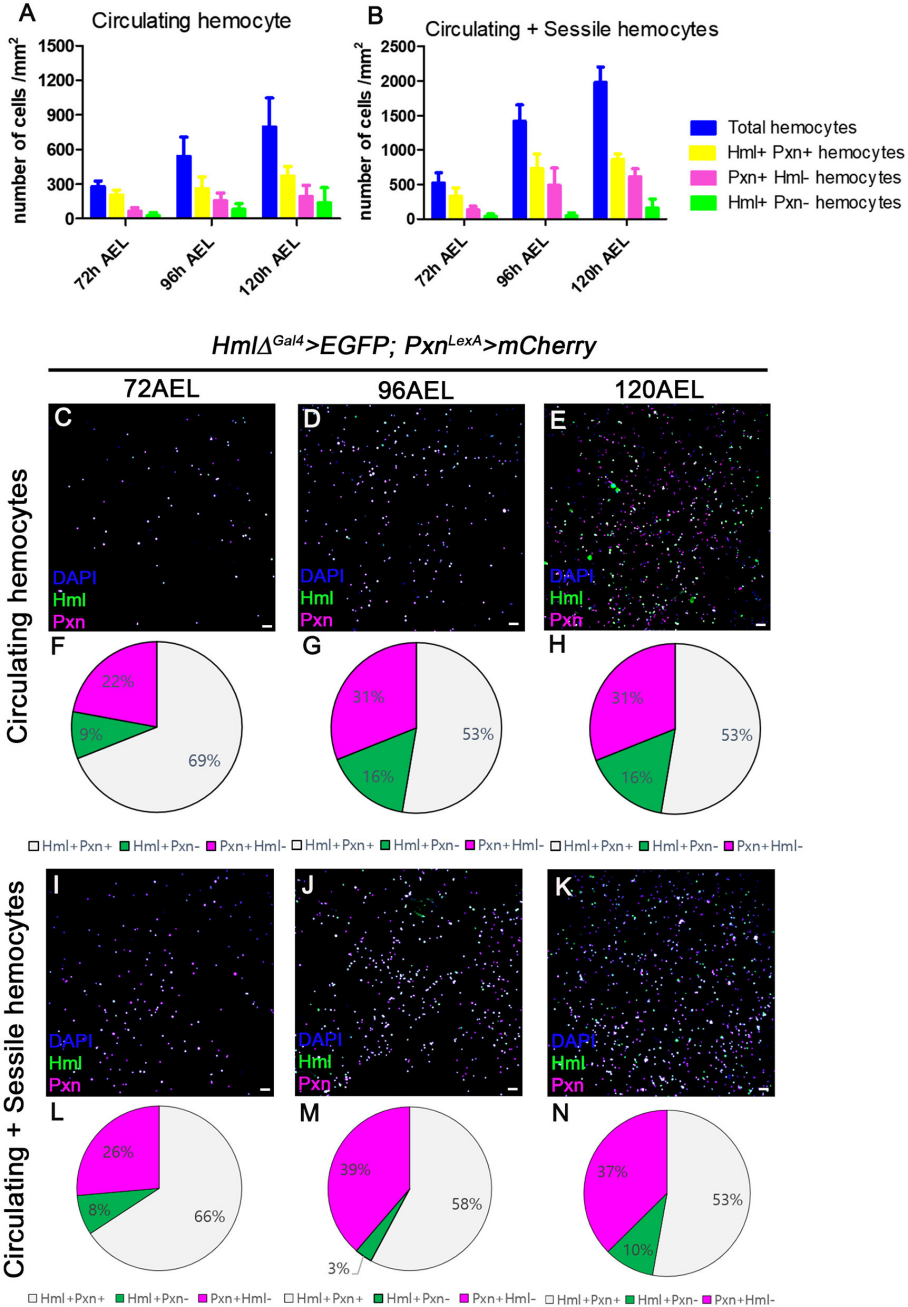
Distribution of total hemocytes during larval development. **(A,B)** Absolute numbers of hemocytes per mm^2^ at 72, 96, and 120 h after egg laying (AEL). Total (blue), *Hml*^+^*Pxn*^+^ (yellow), *Pxn*^+^*Hml*^−^ (magenta), and *Hml*^+^*Pxn*^−^ (green) hemocytes are increased over development in circulating **(A)** and total hemocytes **(B)**. Total hemocytes are counted after vortexing larvae, therefore, indicating sessile and circulating populations (54). Error bars indicate S.D. **(C–E)** Circulating hemocytes bled at 72 **(C)**, 96 **(D)**, and 120 h AEL **(E)**. Subtypes of plamatocytes are visualized by *Hml* (green), *Pxn* (magenta), and DAPI (blue) (*Hml*^Δ^*-Gal4 UAS-EGFP; Pxn-LexA LexAop-mCherry*). Co-localization of *Hml* and *Pxn* is indicated in white. Scale bar, 40 μm. **(F–H)** Pie chart quantitation of circulating plasmatocytes at 72 (**F**, related to **C**), 96 (**G**, related to **D**), and 120 h AEL (**H**, related **E**). *Hml*^+^*Pxn*^+^ (white), *Pxn*^+^*Hml*^−^ (magenta), and *Hml*^+^*Pxn*^−^ (green). **(I–K)** Circulating and sessile hemocytes bled at 72 **(I)**, 96 **(J)**, and 120 h AEL **(K)**. Subtypes of plamatocytes are visualized by *Hml* (green), *Pxn* (magenta), and DAPI (blue) (*Hml*^Δ^*-Gal4 UAS-EGFP; Pxn-LexA LexAop-mCherry*). Co-localization of *Hml* and *Pxn* is indicated in white. Scale bar, 40 μm. **(L–N)** Pie chart quantitation of circulating and sessile plasmatocytes at 72 (**L**, related to **I**), 96 (**M**, related to **J**), and 120 h AEL (**N**, related **K**). *Hml*^+^*Pxn*^+^ (white), *Pxn*^+^*Hml*^−^ (magenta), and *Hml*^+^*Pxn*^−^ (green).

Using multiple binary systems including the Gal4-UAS and LexA-LexAop, we can simultaneously visualize several genetic markers and study interactions of respective genes or tissues (47, 48, 53). Expression of *Hml* or *Pxn* have been exploited to illuminate our understandings on development and functions of plasmatocytes, and utilized as markers for plasmatocytes across several *Drosophila* hematopoiesis analyses (20). However, it remains unclear whether these markers equally label entire plasmatocytes in larval circulation at the transcript level. To understand discrepancies between the two markers in larval hemocytes, we generated *Pxn-LexA* construct to concurrently visualize *Hml* and *Pxn* in larval hemocytes (refer to method for details). By utilizing two binary systems, we first verified the distribution of *Hml*^+^ and *Pxn*^+^ populations in embryonically derived hemocytes at 72, 96, and 120 h after egg laying (AEL) (Figures 1A,B). With the circulating portion of hemocytes, we observed more than 50% overlap between *Hml*^+^ and *Pxn*^+^ plasmatocytes at each time point, though specific ratios differ (Figures 1C–H). Total hemocytes including circulating and sessile populations show similar proportions of *Hml*^+^ and *Pxn*^+^ plasmatocytes (Figures 1I–N). Besides the *Hml* and *Pxn* double-positive plasmatocytes, *Hml*-positive and *Pxn*-negative (*Hml*^+^ *Pxn*^−^) or *Pxn*^+^ *Hml*^−^ subpopulations are indicated in both circulation and total hemocyte samples (Figures 1F–H,L–N). Though the larger of the two subpopulations—*Pxn*^+^ *Hml*^−^ and *Hml*^+^ *Pxn*^−^–is *Pxn*^+^ *Hml*^−^, both *Hml*^+^ *Pxn*^−^ and *Pxn*^+^ *Hml*^−^ are distinct at all-time points (Figures 1F–H,L–N). Staining for P1, a mature plasmatocyte marker, hnt, a crystal cell marker or L1, a lamellocyte marker, shows that both subpopulations are randomly co-localized with P1 and hnt (Supplementary Figures 1A–I). L1 does not show any expression under normal culture conditions in larval plasmatocytes (Supplementary Figures 1G–I) These patterns suggest that plasmatocyte subtypes expressing *Pxn*^+^ *Hml*^−^ and *Hml*^+^ *Pxn*^−^ are not exclusive to crystal cells nor late plasmatocytes, and are not lamellocytes.

### Distribution of *Pxn*^+^ or *Hml*^+^ Hemocytes Are Changed by Physiological Alterations

Hemocyte population size is known to be influenced by changes in internal and external conditions (12, 46, 55). Based on the differential expressions of *Pxn* and *Hml* in larval hemocytes, we next sought to understand whether the ratios of the plasmatocyte subpopulations seen under normal growing conditions can be adjusted by distinctive physiological conditions. We designed physiological challenges in 2-folds: immunological and metabolic, given previous notions linking immunity and metabolism (42, 45). First, we infested the second-instar larvae at 60 h AEL by wasps (*Leptopilina boulardi*) and examined expressions of *Pxn*^+^ or *Hml*^+^ hemocytes at 72, 96, and 120 h AEL. Interestingly, we observe that wasp infestation induces a biased expansion of *Pxn*^+^ *Hml*^−^ hemocytes from 12 h post infestation (PI; 72 h AEL) prior to the massive proliferation of total hemocytes (Supplementary Figures 1J,M). Moreover, the proportion of *Pxn*^+^ *Hml*^−^ hemocytes further expands along with excessive proliferation of total hemocytes and differentiation of lamellocytes at 96 and 120 h AEL (Supplementary Figures 1K,L,N,O). Second, we modified internal metabolism by supplying additional sucrose to the normal diet (56). Similar to the immunological challenge, we found an increase in *Pxn*^+^ *Hml*^−^ hemocytes after chronic supplementation of high sucrose diet (Supplementary Figures 1P–U). Distinct from the wasp infestation, high sucrose diet does not increase *Pxn*^+^ *Hml*^−^ hemocytes from 72 h AEL; however, the percentage of *Pxn*^+^ *Hml*^−^ hemocytes is drastically augmented by 120 h AEL (Supplementary Figures 1R,U). These results indicate that proportions of *Pxn*^+^ *Hml*^+^, *Pxn*^+^ *Hml*^−^ or *Hml*^+^ *Pxn*^−^ hemocytes are plastic and amenable upon an immune challenge generated by wasp infestation or a metabolic alteration induced by supplementation of high sugar diet. Also, these physiological changes readily alter plasmatocytes ratios seen under normal culture conditions, driving a biased expansion of *Pxn*^+^ *Hml*^−^ hemocytes.

### Hemocytes Produce High Levels of *upd3* in the Absence of *Hml*^+^ Cells

**Figure 2:**
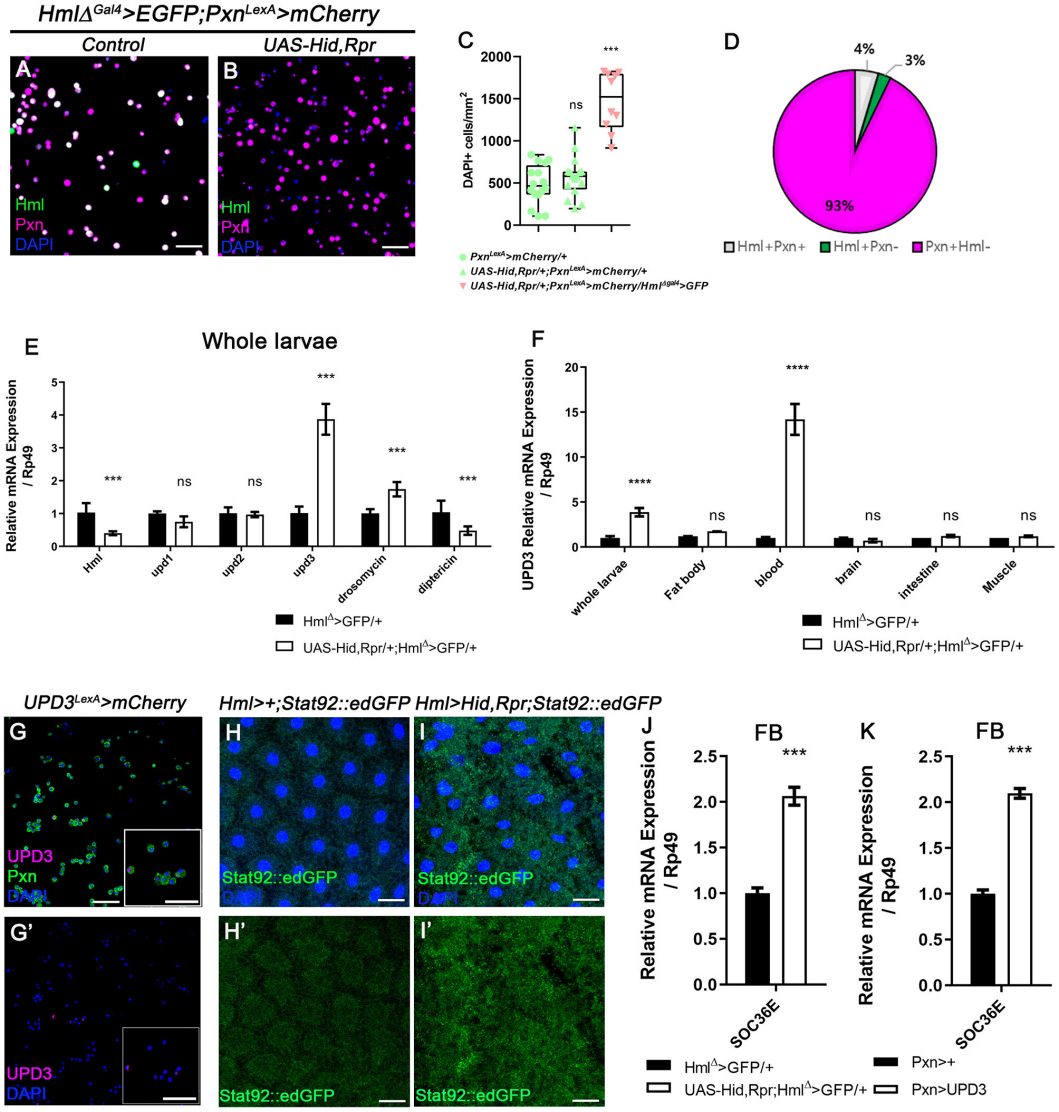
Ablation of *Hml*^+^ hemocytes increases the number of circulating and sessile hemocytes, and induces *upd3* expression. **(A,B)** Expression of *Pxn* (magenta) in *Hml*^Δ^*-Gal4 UAS-EGFP, Pxn-LexA LexAop-mCherry* background. Compared to hemocytes sampled from wild type **(A)**, the genetic ablation of *Hml*^+^ cells increases the number of *Pxn*^+^ (magenta) cells (*Hml*^Δ^*-Gal4 UAS-EGFP UAS-hid,rpr, Pxn-LexA LexAop-mCherry*) **(B)**. *Hml* (green), *Pxn* (magenta), and DAPI (blue). Scale bar, 40 μm. **(C)** Quantitation of circulating and sessile hemocytes in genetic backgrounds used in **(A,B)**. Graphs indicate median plots of DAPI positive cells per mm^2^ in each genotype. Highest and lowest bars indicate maximum and minimum values, respectively. Controls (green) (*Pxn-LexA LexAop-mCherry* or *UAS-hid,rpr; Pxn-LexA LexAop-mCherry*), *Hml* ablated background (pink) (*Hml*^Δ^*-Gal4 UAS-EGFP UAS-hidrpr; Pxn-LexA LexAop-mCherry*). Statistical significance was determined by *t-test*. ^***^p < 0.001; not-significant, ns. **(D)** Pie chart shows the proportion of *Hml*^+^*Pxn*^+^ (white), *Hml*^+^*Pxn*^−^ (green), or *Pxn*^+^*Hml*^−^ (magenta) in circulating and sessile plasmatocytes of *Hml*^+^ablated background (*Hml*^Δ^*-Gal4 UAS-EGFP UAS-hidrpr; Pxn-LexA LexAop-mCherry*). Quantitation of **(B)**. **(E,F)** mRNA levels of signaling molecules and antimicrobial peptides in *Hml*^+^ablated background (*Hml*^Δ^*-Gal4 UAS-hid,rpr*). RT-qPCR analysis of genes related to active immunity normalized by *rp49* using whole larvae **(E)**. *upd3* expression in relevant organs **(F)**. *upd3* expression is highly increased in whole larvae **(E)**, and hemocytes show identical increase in *upd3* **(F)**. Statistical analyses were performed using two-way ANOVA in whole larvae and organs. ^***^p < 0.001; ^****^p < 0.0001; not significant, ns. **(G,G')** *upd3* (magenta; *upd3-LexA LexAop-mRFP*) is co-localized with Pxn (green) in total hemocytes. Inset in magnified view. DAPI is blue. Scale bars, 40 μm. **(H,H',I,I')** *STAT92E::edGFP* (green) expression is increased in the fat body upon loss of *Hml*^+^ hemocytes. **(H,H')** is control (*Hml-Gal4; STAT92E::edGFP*) and **(I,I')** is *Hml*^+^ ablated background (*Hml-Gal4 UAS-hid, rpr; STAT92E::edGFP*). Scale bars, 80 μm. **(J,K)** *Socs36E* is increased in the fat body when *Hml*^+^ hemocytes are genetically ablated or *upd3* is ectopically expressed in *Pxn*^+^ cells. mRNA expression of *Socs36E* is increased in the fat body extracted from *Hml*^Δ^*-Gal4 UAS-hid, rpr* **(J)** or from *Pxn-gal4 UAS-upd3* **(K)**. Graph indicates RT-qPCR analyses of *Socs36E* in in the fat body. Error bar in graph is S.D. Statistical significance was determined by using *t-test*. ^***^p < 0.001.

Our observations indicate that *Pxn*^+^ *Hml*^−^ plasmatocytes are the second largest plasmatocyte subtype comprising ~30% of total hemocytes and modified upon immune or nutritional insults. Despite the relatively large proportion of *Pxn*^+^ *Hml*^−^ hemocytes in the plasmatocyte population, the function of this subtype has not been explored. Thus, we next examined the expression and possible functions of *Pxn*^+^ *Hml*^−^ plasmatocytes by reducing the *Hml*^+^ population. First, we genetically ablated the *Hml*^+^ hemocytes by expressing proapoptotic genes, *hid* and *reaper* (*rpr*) from the first instar of larval development (*Hml*^Δ^*-Gal4, UAS-hid, rpr*), and observed changes in the numbers and proportions of total hemocytes in circulation (Figures 2A,B, Supplementary Figures 2A–D). Compared to controls, the *Hml* ablated background shows four times increment in the total hemocyte count (Figure 2C, Supplementary Figure 2E). Associated with this phenotype, the *Pxn*^+^ *Hml*^−^ subpopulation expands to as much as 93% of the total plasmatocytes (Figure 2D). Moreover, the number of lamellocytes is increased comparable to previously reported phenotype (Supplementary Figures 2F,G) (57). We verified that the remaining cells upon loss of *Hml*^+^ hemocytes express *Pxn* (Supplementary Figures 2H,I). Interestingly, the remainder *Pxn*^+^ plasmatocytes do not show *Hml* expression and are not apoptotic (Supplementary Figures 2J–L,J',K'), demonstrating that these plasmatocytes are not *Hml*^+^ nor dying cells, but expressing *Pxn*. We repeated this experiment by temporarily ablating *Hml*^+^ hemocytes only at the third-instar stage. However, the acute elimination further reduces the number of total hemocytes while concomitantly increasing caspase-positive cells (Supplementary Figures 2M–P,M',N'), demonstrating that temporal expression of *hid* and *rpr* in *Hml*^+^ hemocytes exerts differential effects to hemocytes and only chronic ablation gives rise to the biased expansion of *Pxn*^+^ *Hml*^−^ cells. As a second approach, we reduced the *Hml*^+^ hemocytes by expressing *Ras85D* RNA*i* in *Hml*^+^ hemocytes (*Hml*^Δ^*-Gal4, UAS-Ras85D RNAi*). Though *Ras85D* RNA*i* significantly reduces the total hemocytes including *Hml*^+^ population, the ratio of plasmatocyte subpopulations is fairly maintained (Supplementary Figures 3A–D). Therefore, we reasoned that the *Pxn* and *Hml* have differential expressions in circulating and sessile hemocytes, and selective reduction in *Hml* subpopulation raises the *Pxn* subtypes in specific conditions.

Overall increase of *Drosophila* hemocyte populations has been attributed to systemic immune signaling (37). To ratify the causal systemic molecule for the upsurge of remnant plasmatocyte upon *Hml*^+^ hemocyte ablation, we performed whole-larva real-time quantitative polymerase chain reaction (RT-qPCR). We targeted two representative antimicrobial peptides, *Drosomycin* and *Diptericin*, and three cytokines–*upd, upd2*, and *upd3*– as putative indicators of immune activation (58, 59). Moreover, we additionally checked PDGF- and VDGF-related factors, *pvf1, pvf2*, and *pvf3*, as markers for hemocyte migration (60). We observed that *Drosomycin* is significantly up-regulated by ablating *Hml*^+^ hemocytes whereas *Diptericin* exhibits a marked decrease (Figure 2E). Remarkably, amongst all the other candidates, *unpaired 3* (*upd3*) is the most excessively induced (Figure 2E, Supplementary Figure 3E). To determine the source of increased *upd3*, we screened the expression of *upd3* in tissues including fat body, hemocytes, brain, intestine and muscle, after removing *Hml*^+^ hemocytes. Notably, we found that hemocytes exclusively produce the highest *upd3* mRNA in the ablated background (Figure 2F). Related to this phenotype, we observed that the increase of *upd3* in hemocytes is recapitulated by feeding a high sucrose diet (Supplementary Figure 3F), implying that the expanded *Pxn*^+^ hemocytes boost *upd3* production independent of the apoptosis of *Hml*^+^ hemocytes. We further verified that upd3 co-localizes with *Pxn*^+^ hemocytes (Figures 2G,G'). On the other hand, expression of *Ras85D* RNA*i* in *Hml*^+^ cells leads to lowering of *upd3* expression in hemocytes, different from *upd3* mRNA expression driven by loss of *Hml*^+^ hemocytes or supplementation of high sucrose diet (Supplementary Figure 3G). Hence, we concluded that *upd3* mRNA is induced in hemocytes upon ablation of *Hml*^+^ hemocytes, possibly due to the expansion of *Pxn*^+^ hemocytes.

### Loss of *Hml*^+^ Hemocytes Alters Systemic Growth of Animal

While observing hematopoietic phenotypes in *Drosophila* larvae, we noticed that animals with *Hml*^Δ^*-Gal4 UAS-hid,rpr* show delayed pupation than wildtype controls (Supplementary Figure 4A). This systemic growth delay prompted us to further quantify pupal and adult growth parameters which can be consequences of imbalanced hemocytes or prolonged expression of a cytokine *upd3* (61). We detected that chronic expression of *hid* and *rpr* in *Hml*^+^ hemocytes during larval stages significantly reduces the size of pupae (Supplementary Figures 4B–D). Consistently, similar reduction is observed in both male and female adult flies (Supplementary Figures 4E–G), suggesting that persistent loss of *Hml*^+^ hemocytes attenuates growth and decreases the size of animals. We next addressed whether the small size of animals is caused by an increase in *upd3* expression, and found that ectopic expression of *upd3* using *Pxn-Gal4* recapitulates the size reduction comparable to that shown in *Hml*^Δ^*-Gal4 UAS-hid,rpr* flies (Supplementary Figure 4H). Overall, we concluded that high levels of *upd3* derived upon loss of *Hml*^+^ hemocytes systemically suppresses animal growth from larvae to adult flies.

### Active JAK/STAT Signaling Attenuates Insulin Pathway in Fat Body

The JAK/STAT pathway is a common downstream target of *upd3*, and is known to be involved in innate immune responses including hemocyte proliferation and lamellocyte differentiation (36, 37). Given that loss of *Hml*^+^ hemocytes induces *upd3* expression in hemocytes, we next examined the activity of JAK/STAT signaling and its downstream target tissue upon loss of *Hml*^+^ hemocytes. Using a *STAT92E::edGFP* reporter (62), we ascertained that four organs—fat body (Figures 2H–I'), muscle (Supplementary Figures 5A–B'), intestine (Supplementary Figures 5C–D') and trachea (Supplementary Figures 5E–F')—exhibit substantially high *STAT92E::edGFP* activities in the *Hml*^+^ hemocyte ablated background. Yet, two organs including the brain and the salivary gland did not show any considerable changes (Supplementary Figures 5G–J'). These patterns are reflected in RT-qPCR analyses using *Socs36E*, a downstream target of JAK/STAT pathway (Supplementary Figure 5K). Among the four organs with high *STAT92E::edGFP* expression, we focused on the fat body considering systemic phenotypes of small animal size and active antimicrobial peptide gene expressions in *Hml*^Δ^*-Gal4 UAS-hid, rpr* background (Supplementary Figures 4A–H, Figure 2E). To add, *Hml*^Δ^*-Gal4 UAS-hid, rpr* shows high level of *Socs36E* in the fat body, comparable to that observed when *upd3* is overexpressed in *Pxn*^+^ hemocytes (Figures 2J,K), confirming that the fat body is indeed stimulated by *upd3* originating from hemocytes upon loss of *Hml*^+^ hemocytes.

**Figure 3:**
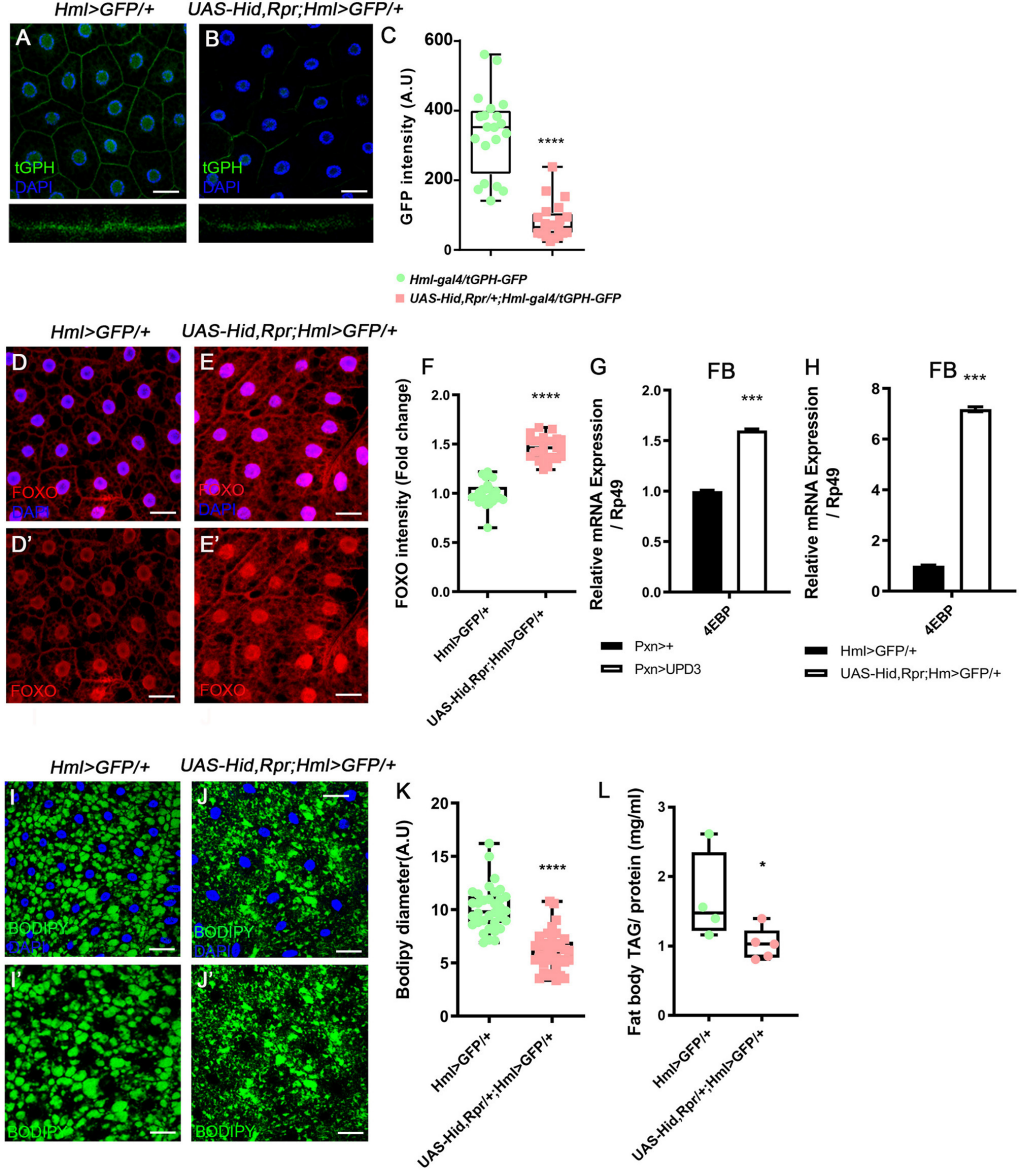
Attenuated insulin signaling in the fat body upon loss of *Hml*^+^ hemocytes. **(A–C)** Membrane localization of *t*GPH, a reporter for PI3K activity, is reduced by ablating *Hml*^+^ hemocytes. Fat body isolated from wild types exhibits membrane-expression of *t*GPH (green) **(A)**, whereas this pattern disappears in *Hml-Gal4 UAS-hid,rpr* background **(B)**. Bottom images indicate magnified Z-stacks of corresponding images. Quantitation of *t*GPH expression in the fat body membrane **(C)**. DAPI, blue. Scale bar, 40 μm. Statistical significance was determined by *t-test*. ^****^p < 0.0001. **(D–F)** Expression of dFOXO upon genetic ablation of *Hml*^+^ hemocytes. Cytosolic and low expressions of dFOXO in the wild-type fat body **(D,D')**. The level and nuclear localization of dFOXO is enhanced in *Hml-Gal4 UAS-hid,rpr* background **(E,E')**. DAPI (blue) and dFOXO (red) are overlaid in **(D,E)**, and dFOXO (red) alone is shown in **(D',E')**. Nuclear dFOXO levels are quantitated in **(F)**. Statistical significance was determined by *t-test*. ^****^p < 0.0001. Scale bar, 40 μm. **(G,H)** mRNA expression of *4EBP* is increased in both *Hml*^Δ^*-Gal4 UAS-EGFP UAS-hidrpr* **(G)** and *Pxn-Gal4 UAS-upd3* **(H)** backgrounds in the fat body. Error bar in graph is S.D. Statistical significance was determined by *t-test*. ^***^p < 0.001. **(I–K)** Expression of lipid droplets in the fat body. Compared to wild types **(I,I')** (*Hml*^Δ^*-Gal4 UAS-EGFP*), the size of lipid droplets is decreased when *Hml*^+^ hemocytes are ablated **(J,J')** (*Hml-Gal4 UAS-hid, rpr*). Quantitation of BODIPY diameter in **(I',J') (K)**. Highest and lowest bars indicate maximum and minimum values, respectively. Statistical significance was determined by *t-test*. ^****^p < 0.0001. Scale bar, 40 μm. **(L)** Biochemical measurement of triacyl glyceride (TAG) levels in the fat body normalized by protein contents. The level of TAG is decreased upon loss of *Hml*^+^ hemocytes (*Hml-Gal4 UAS-hid, rpr*). Highest and lowest bars indicate maximum and minimum values, respectively. Statistical significance was determined by *t-test*. ^*^p < 0.05.

Following the metabolic phenotypes of *Hml* ablated animals, we hypothesized that increased *upd3* obstructs insulin signaling, the representative signaling for systemic growth, in the fat body. To understand whether insulin signaling in the fat body is directly altered by loss of *Hml*^+^ hemocytes, we examined the expression of tGPH, a PI3K reporter (63), and observed that membrane localization of tGPH is diminished in the fat body (Figures 3A–C). This data indicates that PI3K is not recruited to the fat body cell membranes, and therefore, not activated in the *Hml* ablated background. PI3K activation delocalizes FOXO from cell nuclei, inhibits 4EBP, and prevents lipolysis (64). However, when *Hml*^+^ hemocytes are ablated, nuclear localization of dFOXO is induced, and a transcriptional target of dFOXO, *4EBP*, is increased (Figures 3D–G). The ascent in *4EBP* in the fat body recurs when *upd3* is overexpressed in *Pxn*^+^ hemocytes (Figure 3H). In addition, fat storage in the fat body is reduced (Figures 3I–L). All of these findings imply that, insulin receptor (InR) signaling is abrogated in the absence of *Hml*^+^ hemocytes. Furthermore, *InR* mRNA in the fat body is decreased when *Hml*^+^ hemocytes are ablated or *upd3* is overexpressed in *Pxn*^+^ hemocytes (Supplementary Figures 5L–M), demonstrating that high levels of *upd3* is sufficient to reduce *InR* mRNA expression in the fat body. Thus, ablation of *Hml*^+^ hemocytes causes an increase in *upd3* expression in hemocytes, and the downregulation of insulin signaling indicated by: reduced PI3K activity, nuclear localization of dFOXO, upregulation of *4EBP*, and reduced triacyl glycerides storage in the fat body.

### *upd3* Is Required for Hemocyte Expansion and Systemic Metabolic Responses

**Figure 4:**
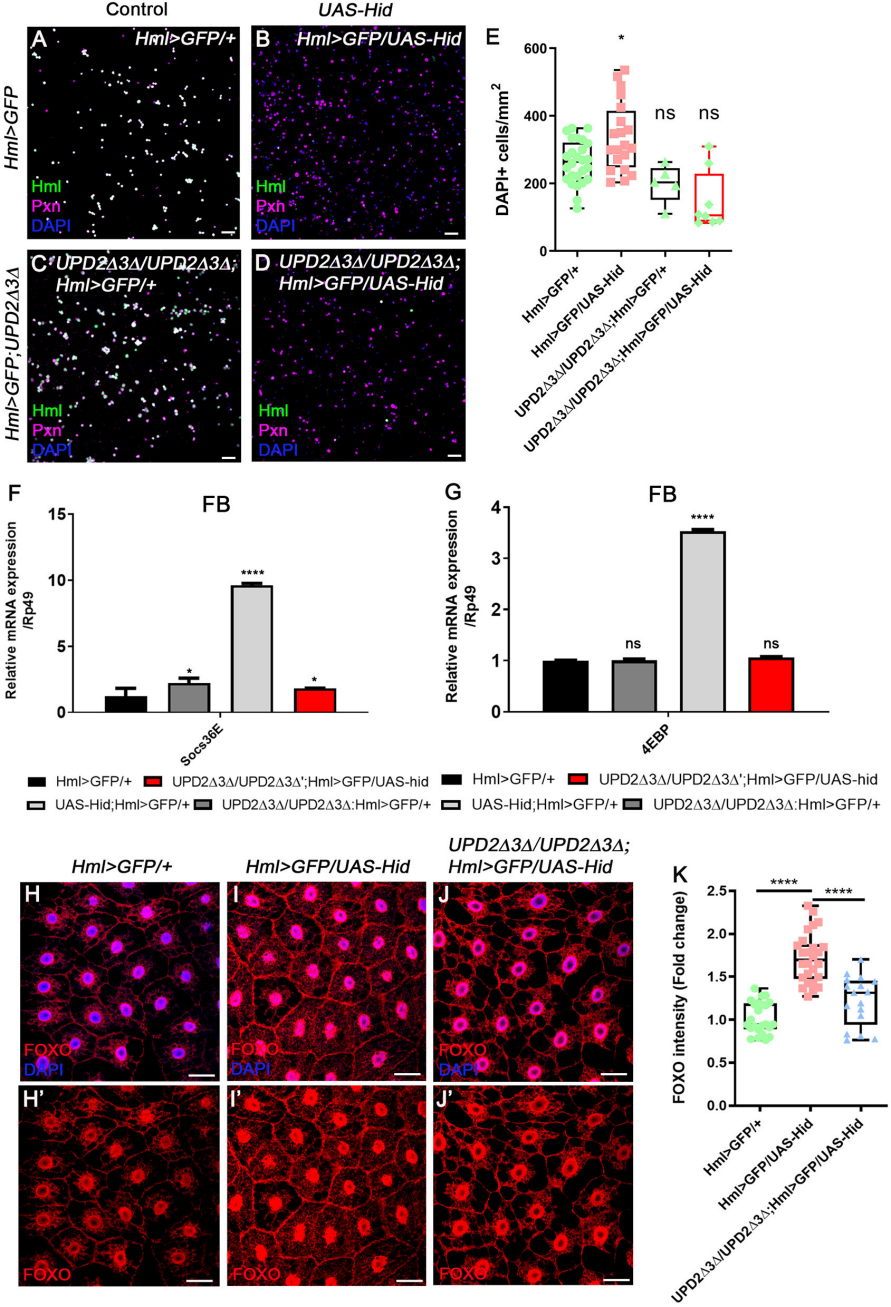
*upd2*^Δ^*upd3*^Δ^ mutants rescue the phenotypes caused by genetic ablation of *Hml*^+^ hemocytes. **(A–E)** The number of total hemocytes is decreased in *upd2*^Δ^*upd3*^Δ^; *Hml-Gal4 UAS-hid* background. Compared to wild types **(A)**, overexpression of *hid* in *Hml*^+^ hemocytes (*Hml-Gal4 UAS-hid*) induces the number of Pxn^+^ (magenta) and/or DAPI^+^ (blue) cells **(B)**. This phenotype is recovered by combining *upd2*^Δ^*upd3*^Δ^ in the *Hml-Gal4 UAS-hid* background **(C,D)**. There is no significant difference between *Hml*^Δ^*-Gal4 UAS-EGFP* controls **(A)** and *upd2*^Δ^*upd3*^Δ^ **(C)** or *upd2*^Δ^*upd3*^Δ^*; Hml-Gal4 UAS-hid* **(D)**. Controls **(A,C)** express *Hml* (green), Pxn (magenta), and DAPI (blue); therefore, are indicated in white. Quantitation of each genotype is shown in **(E)**. Graphs indicate median plots of DAPI positive cells per mm^2^ in each genotype. Highest and lowest bars indicate maximum and minimum values, respectively. Statistical significance was determined by *t-test*. ^*^p < 0.05; not significant, ns. Scale bar, 40 μm. **(F,G)** Increased levels of *Socs36E* or *4EBP* are reverted by loss of *upd2* and *upd3*. Increased levels of *Socs36E* **(F)** or *4EBP* **(G)** upon loss of *Hml*^+^ hemocytes in the fat body are recovered in *upd2*^Δ^*upd3*^Δ^; *Hml-Gal4 UAS-hid* genetic background. Rescue is indicated in red. Statistical analyses were performed using two-way ANOVA. ^*^p < 0.05; ^****^p < 0.0001; not significant, ns. **(H–K)** Nuclear location and levels of dFOXO in the fat body are rescued by loss of *upd2* and *upd3*. In controls, low levels of dFOXO (red) are located in the cytoplasm and nucleus **(H,H')**. Overexpression of *hid* in *Hml*^+^ hemocytes further induces the nuclear expression of dFOXO (red) in the fat body **(I,I')**. Genetic combination of *upd2*^Δ^*upd3*^Δ^ and *Hml-Gal4 UAS-hid* reduces the nuclear expression of dFOXO (red) **(J,J')**. Quantitation of dFOXO intensity shown in **(H–J') (K)**. Highest and lowest bars indicate maximum and minimum values, respectively. Statistical significance was determined by *t-test*. ^****^p < 0.0001. Scale bar, 40 μm.

Given our new findings that enhanced expression of *upd3* upon loss of *Hml*^+^ hemocytes abrogates systemic growth through insulin signaling, and JAK/STAT activities in the fat body, we next investigated whether *upd3* is solely responsible for the phenotypes—hemocyte proliferation, active JAK/STAT signaling in the fat body, and altered metabolism of animals—shown in *Hml*^+^ ablated backgrounds. Since both *upd2*^Δ^*3*^Δ^ genes and *UAS-hid,rpr* transgenes are localized in the first chromosome, we utilized an alternative transgene, *UAS-hid* to combine all the genotypes. The number of circulating and sessile plasmatocytes as well as lamellocytes are greatly increased when *hid* and *rpr* are overexpressed in *Hml*^+^ hemocytes (Figure 2C, Supplementary Figures 2E–G). These phenotypes recur when only *UAS*-*hid* is highly expressed in *Hml*^+^ hemocytes (*Hml*^Δ^*-Gal4 UAS-hid*) (Figures 4A,B,E, Supplementary Figures 6A–E). Also, the remaining *Pxn*^+^ hemocytes do not show *Hml* expression and are not apoptotic when Hml+ hemocytes are ablated in a chronic manner (Supplementary Figures 6F–H). Nonetheless, when *hid* is overexpressed in the *Hml*^+^ hemocytes in *upd2*^Δ^*3*^Δ^ null mutant background (*upd2*^Δ^*upd3*^Δ^*; Hml*^Δ^*-Gal4 UAS-hid*), the numbers of circulating and sessile hemocytes and lamellocytes are restored (Figures 4C–E, Supplementary Figures 6I–K). In the lymph gland, expression of *hid* in *Hml*^+^ hemocytes accelerates precocious differentiation and disintegrates the primary lobe similar to previous observations (Supplementary Figures 6L–M) (65). However, contrary to the circulating and sessile hemocytes, the lymph gland phenotypes are not recovered in *upd2*^Δ^*3*^Δ^ null mutant background (Supplementary Figure 6N). In addition to rescue of blood phenotypes, both the insulin- and JAK/STAT-related phenotypes are rescued when *hid* is overexpressed in *Hml*^+^ hemocytes in the *upd2*^Δ^*3*^Δ^ mutant. First, we noticed that both *Socs36E*, a hallmark for the JAK/STAT signaling, and *4EBP*, a downstream target of insulin pathway, are recovered to normal levels in the fat body when *upd2* and *upd3* are deleted (Figures 4F,G). Second, we confirmed that dFOXO expression is delocalized from fat body nuclei when *UAS-hid* is expressed in *Hml*^+^ hemocytes in the *upd2*^Δ^*3*^Δ^ null mutant background (Figures 4H–K). Altogether, we conclude that high *upd3* induced by ablation of *Hml*^+^ hemocytes is required for the increase in JAK/STAT and decrease in insulin signaling in the fat body, contributing to systemic growth retardation phenotypes in animals.

## Discussion

In this study, we reiterate the heterogeneity of plasmatocyte populations in embryonically derived hemocytes by taking advantage of two distinct binary systems, Gal4-UAS and LexA-LexAoP. In an effort to uncover subpopulations of plasmatocytes, we utilized two representative markers, *Hml* and *Pxn*, and simultaneously measured their transcriptional activities in the embryonically derived hemocytes (20). In both circulation and at sessile sites under normal growing conditions, the proportions of *Pxn*^+^ *Hml*^+^, *Pxn*^+^ *Hml*^−^, or *Hml*^+^ *Pxn*^−^ plasmatocytes are relatively fixed. Moreover, expansion of each subtype is tightly regulated during development suggesting that the composition of plasmatocyte is not random, but, rather controlled. Generally, *Pxn*^+^ *Hml*^+^ hemocytes comprise the largest amongst three subtypes, and *Hml*^+^ *Pxn*^−^ hemocytes represent the population with the least frequency. A slight reduction in *Hml*^+^*Pxn*^−^ hemocytes is observed at 96 h AEL, which could be ascribed to a drastic expansion of other populations including *Pxn*^+^*Hml*^+^ and *Pxn*^+^ *Hml*^−^ during that specific time point (Figures 1J,M). This notion is reinforced by the ability of hemocytes to differentiate or proliferate at sessile sites (12, 26, 66).

When we reared larvae on high sugar diet or infested larvae with wasps, we observed a biased increment of *Pxn*^+^ *Hml*^−^ plasmatocyte subtype, identical to the phenotype observed by ablating *Hml*^+^ hemocytes (Supplementary Figures 1J–U). These data indicate that plasmatocyte subtypes naturally fluctuate according to developmental timings, alterations in nutrition or elevation of innate immunity. Also, the presence of natural plasticity in hemocyte populations during the larval life cycle, and its correspondence to both internal and external environmental changes is asserted. Additionally, the inherent heterogeneity indicates functional divergence of plasmatocytes: the *Hml*^+^ and *Pxn*^+^ population may be progenitor-like and possibly permit trans-differentiation or proliferation; but, *Hml*^+^ *Pxn*^−^ or *Pxn*^+^ *Hml*^−^ subtypes could be specialized to fine-tune different aspects of homeostasis including immunity or metabolism.

In mammals, several studies have verified disparity in macrophage populations, which is captured in M-1 or M-2 macrophages. While M-1 macrophages are prone to reinforce classical immunological phagocytosis behaviors, M-2 macrophages are metabolism biased (67, 68). Similar to the M-1/M-2 macrophages, we expect that there are functional segregations within the plasmatocyte population. It is likely that *Pxn*^+^ *Hml*^−^ population respond to metabolic alterations such as high sucrose diet, and that *Hml*^+^ population is responsible for proper immune responses given lack of lamellocytes caused by reducing the number of *Hml*^+^ hemocytes (Supplementary Figures 6O–Q). These details suggest imaginable conservation of the M-1/M-2 macrophage paradigm in *Drosophila* plasmatocytes, and we expect that plasmatocytes can be further classified by their RNA or protein expression at single-cell resolutions.

Rise in systemic *upd3* is associated with innate immune responses (37). We observed heightened *upd3* expression when *Hml*^+^ hemocytes are depleted. However, considering overall decrease in animal sizes and metabolic responses mediated by the fat body, we assume that the changes in *upd3* in this context is not necessarily immunological. This assertion is consistent with findings from other studies showing that loss of *Hml*^+^ hemocytes leads to muscle degeneration, developmental, and leg defects (57). These non-immune phenotypes may result from increased *upd3*. We expect that the *Pxn*^+^ *Hml*^−^ remnant plasmatocyte population or *Hml*^+^ dying hemocytes are the potential origin of *upd3*. Given that feeding high sucrose diet causes an expansion of *Pxn*^+^ *Hml*^−^ hemocytes and also significantly upregulates the level of *upd3*, we assume that *Pxn*^+^ *Hml*^−^ hemocytes could be a more likely source of the systemic *upd3* (Supplementary Figures 1R,U, 3F). Determining the definitive source of *upd3* among the plasmatocyte subtypes in this condition requires further research.

**Figure 5:**
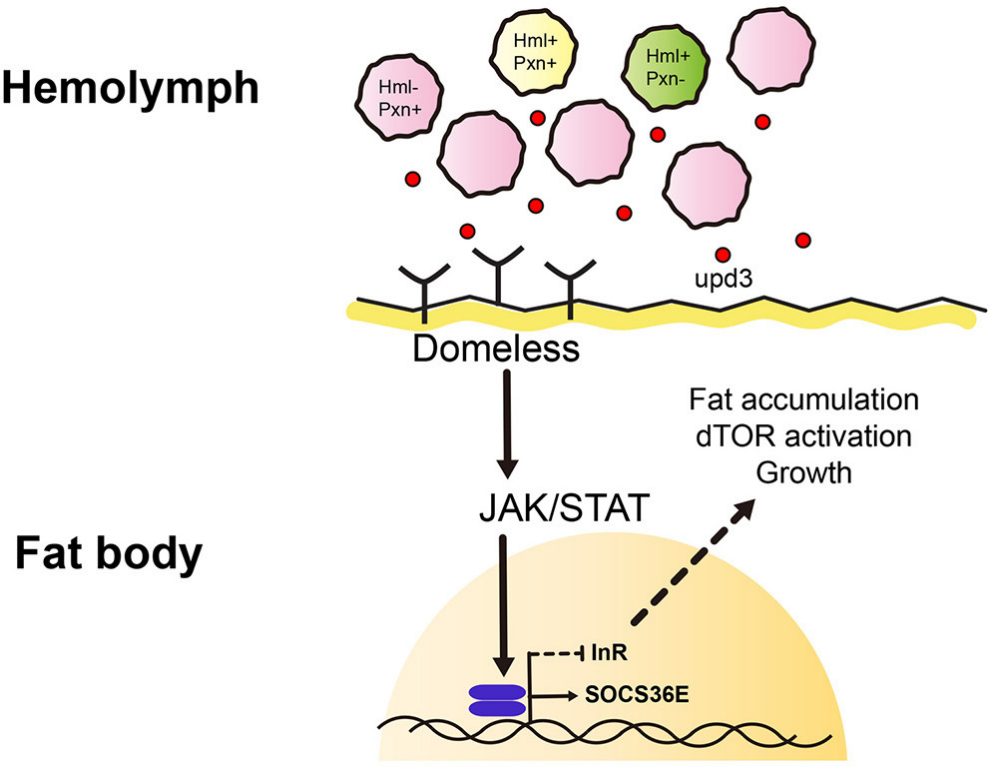
Working model of this study. We identified heterogenic expression of larval plasmatocytes based on two genetic markers, *Hemolectin* (*Hml*) and *Peroxidasin* (*Pxn*). There are *Hml*^+^ *Pxn*^+^ (yellow), *Hml*^+^ *Pxn*^−^ (green), and *Pxn*^+^ *Hml*^−^ (pink) plamatocytes which show definitive distributions across 72, 96, 120 h AEL developmental time points. This suggests plasmatocyte proportions are constantly maintained during development. *Pxn*^+^ hemocyte (pink) is increased when *Hml*^+^ hemocytes are ablated. Also, total remaining hemocytes emit a cytokine, upd3 (red dot). upd3 increases JAK/STAT signaling in the fat body which possibly inhibits insulin receptor signaling, and consequently affects growth and metabolism.

Recent publications have highlighted genetic interactions between the JAK/STAT and insulin signaling as well as putative coupling of immune and metabolic functions (69). Other studies have shown an identical interaction between the JAK/STAT and insulin signaling in the muscle whose interaction is essential for cellular immune responses including lamellocyte differentiation (38, 70). Our study represents an *in vivo* interaction between JAK/STAT and insulin signaling pathways in the fat body (Figure 5). Given that both proximal and distal components of insulin signaling—PI3K and InR respectively—are altered when JAK/STAT pathway is activated, convergence of the two pathways could occur at the direct downstream of InR activity or *InR* transcription level. It will be intriguing to uncover the exact confluence between the two pathways despite the dichotomy that exists between them: while insulin signaling is growth and proliferation-biased, JAK/STAT is immunological (71–74). More so, this may provide insights into how immunity and metabolism differentially interact in normal development and pathologies.

## Data Availability Statement

All datasets generated for this study are included in the article/Supplementary Material.

## Author Contributions

MS, NC, FK, and BC performed experiments. MS, NC, FK, and JS analyzed data and wrote the manuscript. JS conceived the idea and supervised the project.

### Conflict of Interest

The authors declare that the research was conducted in the absence of any commercial or financial relationships that could be construed as a potential conflict of interest.
