# Supplementary material for: Subpopulation of Macrophage-Like Plasmatocytes Attenuates Systemic Growth via JAK/STAT in the Drosophila Fat Body
Source: Front Immunol. 2020 Jan 31;11:63. doi: 10.3389/fimmu.2020.00063 (PMC7005108; doi:10.3389/fimmu.2020.00063)
Supplement: Supplementary file 1 [file Table_1.docx]

**Supplementary Table 1**

| rp49-F | GGC CCA AGA TCG TGA AGA AG |
| --- | --- |
| rp49-R | ATT TGT GCG ACA GCT TAG CAT ATC |
| Hml-F | GTAAGGGTCCCAACTGCGTA |
| Hml-R | CTGGAATGTGTGGACACCAG |
| Diptericin-F | ACCGCAGTACCCACTCAATC |
| Diptericin-R | ACTTTCCAGCTCGGTTCTGA |
| Drosomycin-F | GTACTTGTTCGCCCTCTTCG |
| Drosomycin-R | ACTTCAGACTGGGGCTGC |
| upd1-F | TCCACACGCACAACTACAAGTTC |
| upd1-R | CCAGCGCTTTAGGGCAATC |
| upd2-F | AGTGCGGTGAAGCTAAAGACTTG |
| upd2-R | GCCCGTCCCAGATATGAGAA |
| upd3-F | TGCCCCGTCTGAATCTCACT |
| upd3-R | GTGAAGGCGCCCACGTAA |
| InR-F | GCCGACATAGTCATGGACCT |
| InR-R | AATAACCGGCAGTCTGGTTG |
| 4EBP-F | TCCTGGAGGCACCAAACTTA |
| 4EBP-R | CCTGGTCCTCAATCTTCAGC |
| Pvf1-F | CTGCAACGTGTACCAGTCGT |
| Pvf1-R | TGGTGCAGCTCTGGTTGTAG |
| Pvf2-F | TGATGGCAAAGGAGGGTATC |
| Pvf2-R | TCCTTTTCATTTGGCAGGTC |
| Pvf3-F | ACCTGTCCGAAGAGCTTTGA |
| Pvf3-R | GGCATTGTAGGATGGCTGTT |
| Socs36E-F | CAGTCAGCAATATGTTGTCG |
| Socs36E-R | ACTTGCAGCATCGTCGCTTC |
