# Supplementary figures and images for: Subpopulation of Macrophage-Like Plasmatocytes Attenuates Systemic Growth via JAK/STAT in the Drosophila Fat Body
Source: Front Immunol. 2020 Jan 31;11:63. doi: 10.3389/fimmu.2020.00063 (PMC7005108; doi:10.3389/fimmu.2020.00063)

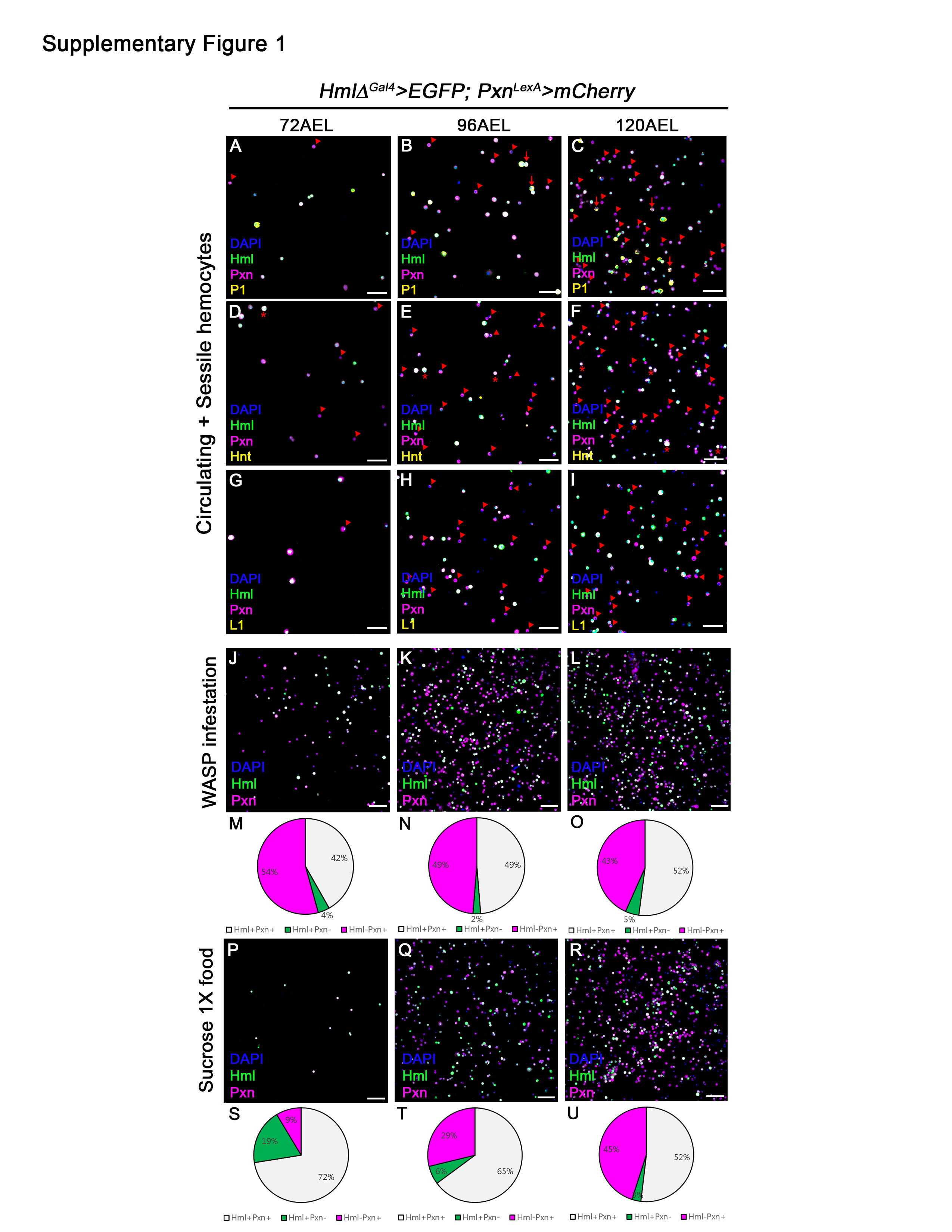

Supplement: Supplementary file 2 [file Image_1.JPEG]

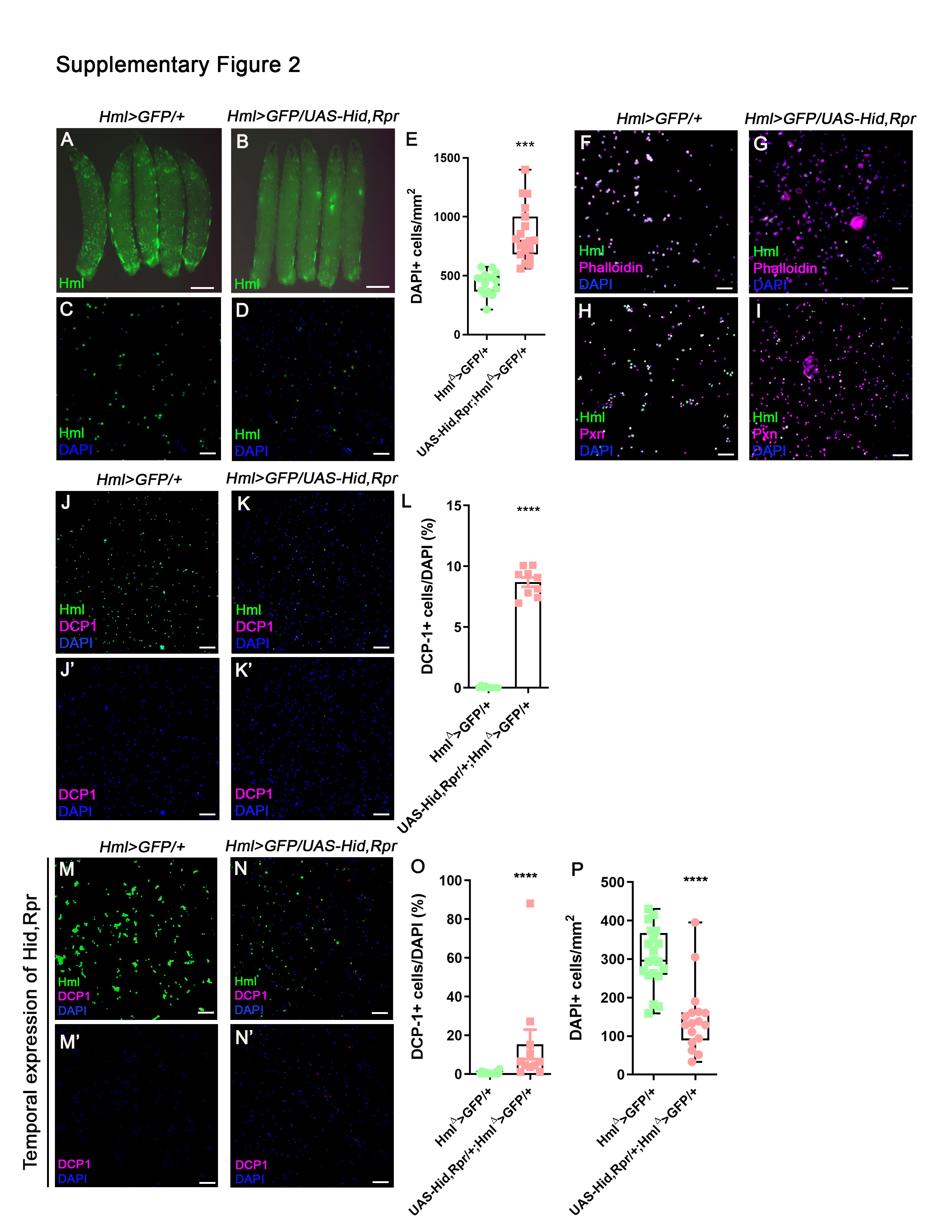

Supplement: Supplementary file 3 [file Image_2.JPEG]

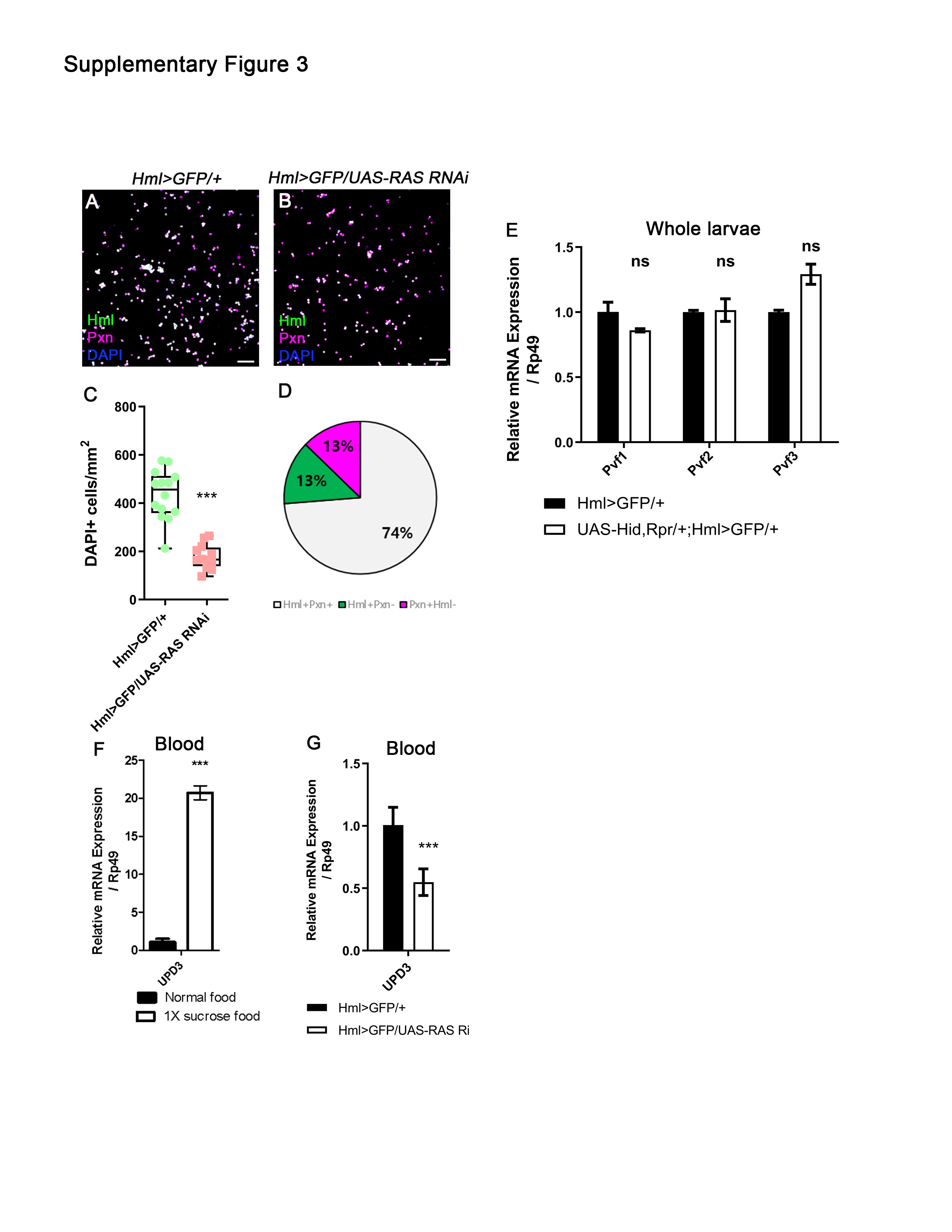

Supplement: Supplementary file 4 [file Image_3.JPEG]

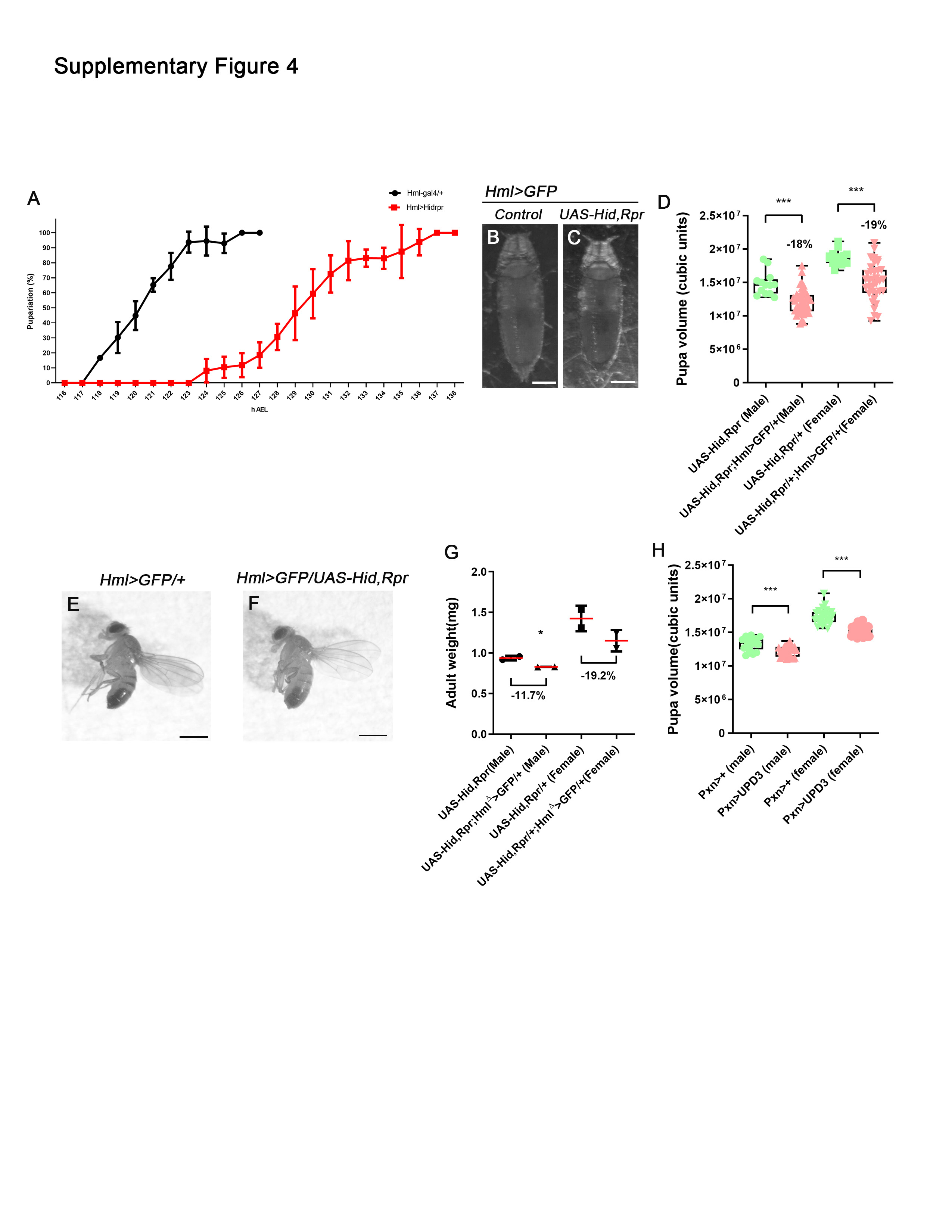

Supplement: Supplementary file 5 [file Image_4.JPEG]

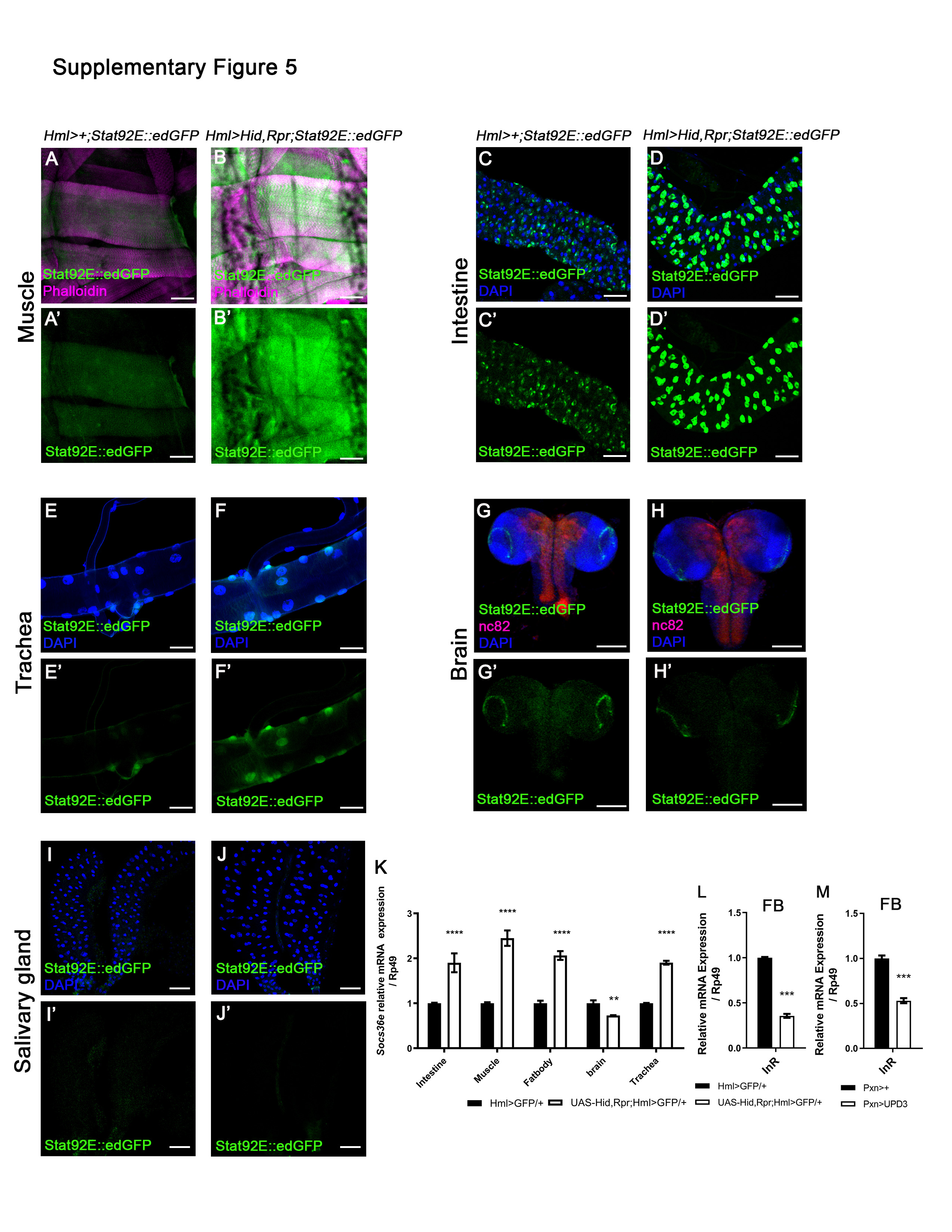

Supplement: Supplementary file 6 [file Image_5.JPEG]

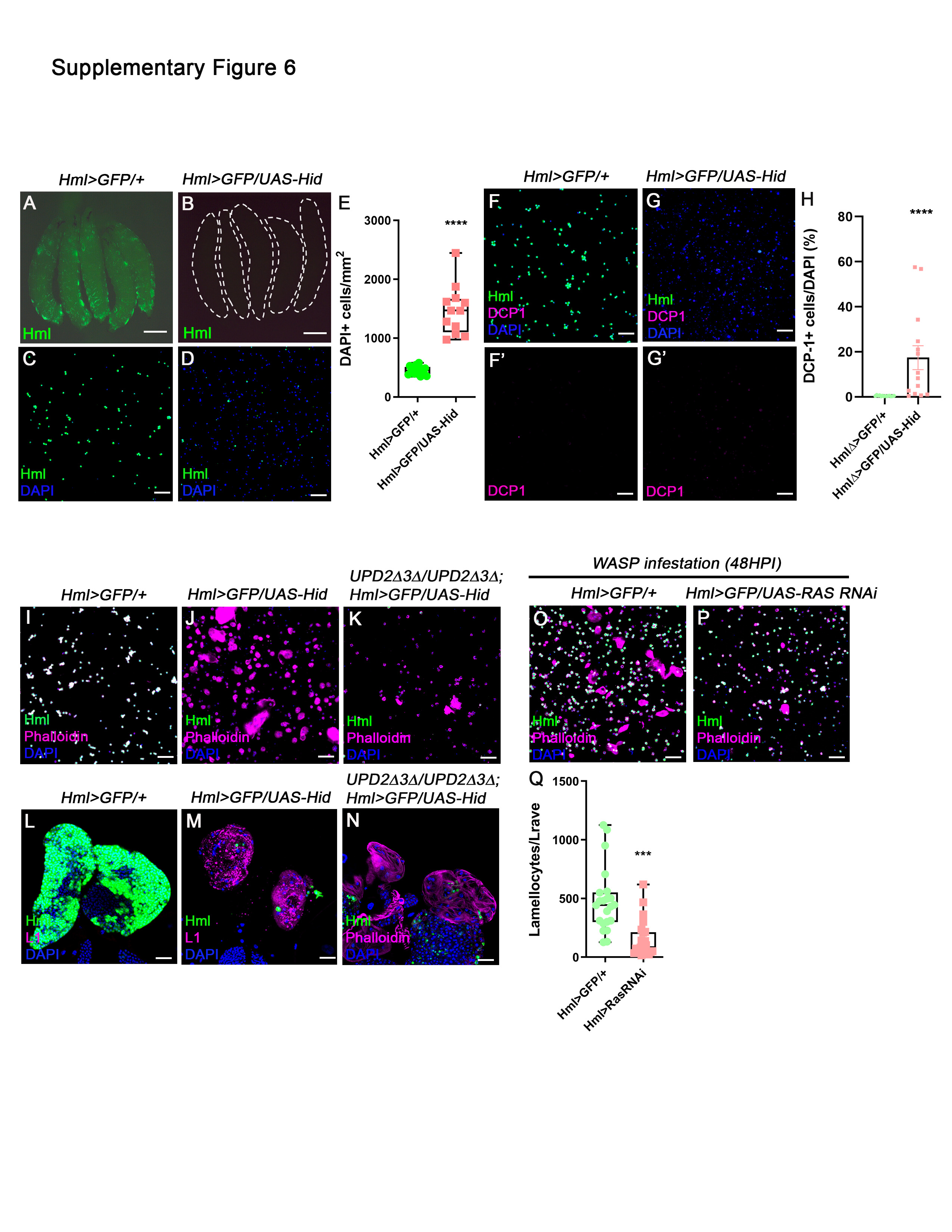

Supplement: Supplementary file 7 [file Image_6.JPEG]
